# Chromosome-scale shotgun assembly using an in vitro method for long-range linkage

**DOI:** 10.1101/gr.193474.115

**Published:** 2016-03

**Authors:** Nicholas H. Putnam, Brendan L. O'Connell, Jonathan C. Stites, Brandon J. Rice, Marco Blanchette, Robert Calef, Christopher J. Troll, Andrew Fields, Paul D. Hartley, Charles W. Sugnet, David Haussler, Daniel S. Rokhsar, Richard E. Green

**Affiliations:** 1 Dovetail Genomics LLC, Santa Cruz, California 95060, USA;; 2 Department of Biomolecular Engineering, University of California, Santa Cruz, California 95066, USA;; 3 UC Santa Cruz Genomics Institute and Howard Hughes Medical Institute, University of California, Santa Cruz, California 95066, USA;; 4 Department of Molecular and Cell Biology, University of California, Berkeley, California 94720, USA;; 5 Department of Energy, Joint Genome Institute, Walnut Creek, California 94598, USA

## Abstract

Long-range and highly accurate de novo assembly from short-read data is one of the most pressing challenges in genomics. Recently, it has been shown that read pairs generated by proximity ligation of DNA in chromatin of living tissue can address this problem, dramatically increasing the scaffold contiguity of assemblies. Here, we describe a simpler approach (“Chicago”) based on in vitro reconstituted chromatin. We generated two Chicago data sets with human DNA and developed a statistical model and a new software pipeline (“HiRise”) that can identify poor quality joins and produce accurate, long-range sequence scaffolds. We used these to construct a highly accurate de novo assembly and scaffolding of a human genome with scaffold N50 of 20 Mbp. We also demonstrated the utility of Chicago for improving existing assemblies by reassembling and scaffolding the genome of the American alligator. With a single library and one lane of Illumina HiSeq sequencing, we increased the scaffold N50 of the American alligator from 508 kbp to 10 Mbp.

A “holy grail” of genomics is the accurate reconstruction of full-length haplotype-resolved chromosome sequences with low effort and cost. High-throughput sequencing methods have sparked a revolution in the field of genomics. By generating data from millions of short fragments of DNA at once, the cost of resequencing genomes has fallen dramatically, rapidly approaching $1000 per human genome (Sheridan 2014). Substantial obstacles remain, however, in transforming short read sequences into long, contiguous genomic assemblies.

Currently accessible and affordable high-throughput sequencing methods are best suited to the characterization of short-range sequence contiguity and genomic variation. Achieving long-range linkage and haplotype phasing requires either the ability to directly and accurately read long (i.e., tens of kilobase) sequences or the capture of linkage and phase relationships through paired or grouped sequence reads.

A number of methods for increasing the contiguity and accuracy of de novo assemblies have recently been developed. Broadly, they attempt either to increase the read lengths generated from sequencing or to increase the insert size between paired short reads that can subsequently be used to scaffold genome assemblies. For example, the PacBio RS II chemistry updated in 2014 is advertised as producing raw reads with mean lengths of 15 kbp but suffers from error rates as high as ∼15% and remains about 100-fold more expensive than high-throughput short reads (Koren et al. 2012; Quail et al. 2012). Commercially available long-reads from Oxford Nanopore are promising but have even higher error rates and lower throughput (Goodwin et al. 2015). These long-read technologies greatly simplify the process of assembly since, in many cases, repetitive or otherwise ambiguous regions of a genome are traversed in single reads. Illumina's TruSeq synthetic long-read technology (formerly Moleculo) is limited to 10-kbp reads maximum (Voskoboynik et al. 2013). CPT-seq is somewhat similar in approach but does not rely on long-range PCR amplification (Adey et al. 2014; Amini et al. 2014). Despite a number of improvements, fosmid library creation (Williams et al. 2012; Wu et al. 2012) remains time-consuming and expensive. To date, the community has not settled on a consistently superior technology for large inserts or long reads that is available at the scale and cost needed for large-scale projects like the sequencing of thousands of vertebrate species (Genome 10K Community of Scientists 2009) or hundreds of thousands of humans (Torjesen 2013).

The challenge of creating reference-quality assemblies from low-cost sequence data is evident in the comparison of the quality of assemblies generated with today's technologies and the human reference assembly (Alkan et al. 2011). Many techniques, including BAC clone sequencing, physical maps, and Sanger sequencing, were used to create the high-quality and highly contiguous human reference standard with an 38.5-Mbp N50 length (the size of the scaffold at which at least half of the genome assembly can be found on scaffolds at least that large) and error rate of one per 100,000 bases (International Human Genome Sequencing Consortium 2004). In contrast, a recent comparison of the performance of whole-genome shotgun (WGS) assembly software pipelines, each run by their developers on very high coverage data sets from libraries with multiple insert sizes, produced assemblies with N50 scaffold length ranging up to 4.5 Mbp on a fish genome and 4.0 Mbp on a snake genome (Bradnam et al. 2013).

High coverage of sequence with short reads is rarely enough to attain a high-quality and highly contiguous assembly. This is due primarily to repetitive content on both large and small scales, including the repetitive structure near centromeres and telomeres, large paralogous gene families like zinc finger genes, and the distribution of interspersed nuclear elements such as LINEs and SINEs. Such difficult-to-assemble content composes large portions of many eukaryotic genomes, for example, 60%–70% of the human genome (de Koning et al. 2011). When such repeats cannot be spanned by the input sequence data, fragmented and incorrect assemblies result. In general, the starting point for de novo assembly combines deep-coverage (50×–200× minimum), short-range (300–500 bp) paired-end “shotgun” data with intermediate range “mate-pair” libraries with insert sizes between 2 and 8 kbp and longer range (35-kbp) fosmid end pairs (Gnerre et al. 2011; Salzberg et al. 2012). However, even mate-pair data spanning these distances is often not completely adequate for generating megabase scale assembles.

Recently, high-throughput short-read sequencing has been used to characterize the three-dimensional structure of chromosomes in living cells. Proximity ligation–based methods like Hi-C (Lieberman-Aiden et al. 2009) and other chromatin capture–based methods (Dixon et al. 2012; Kalhor et al. 2012) rely on the fact that, after fixation, segments of DNA in close proximity in the nucleus are more likely to be ligated together, and thus sequenced as pairs, than are distant regions. As a result, the number of read pairs between intrachromosomal regions is a slowly decreasing function of the genomic distance between them. Several approaches have been developed that exploit this information for the purpose of genome assembly scaffolding and haplotype phasing (Burton et al. 2013; Kaplan and Dekker 2013; Selvaraj et al. 2013; Marie-Nelly et al. 2014).

While Hi-C and related methods can identify biologically mediated long-range chromatin contacts at multi-megabase length scales, most of the data describe DNA–DNA proximity on the scale of tens or hundreds of kilobases. These contacts arise from the polymer physics of the nucleosome-wound DNA fiber rather than from chromatin biology. In fact, the large-scale organization of chromosomes in nuclei provides a confounding signal for assembly since, for example, telomeres of different chromosomes are often associated in cells.

We demonstrate here that DNA linkages up to several hundred kilobases can be produced in vitro using reconstituted chromatin rather than living chromosomes as the substrate for the production of proximity ligation libraries. The resulting libraries share many of the characteristics of Hi-C data that are useful for long-range genome assembly, including a regular relationship between within–read pair distance and read count. By combining this in vitro long-range linking library with standard WGS and jumping libraries, we generated a de novo human genome assembly with long-range accuracy and contiguity comparable to more expensive methods for a fraction of the cost and effort. This method, called “Chicago,” depends only on the availability of modest amounts of high-molecular-weight DNA and is generally applicable to any species. Here we demonstrate the value of this Chicago data not only for de novo genome assembly using human and alligator but also as an efficient tool for the identification and phasing of structural variants.

## Results

### Libraries and sequencing

**Figure 1.**
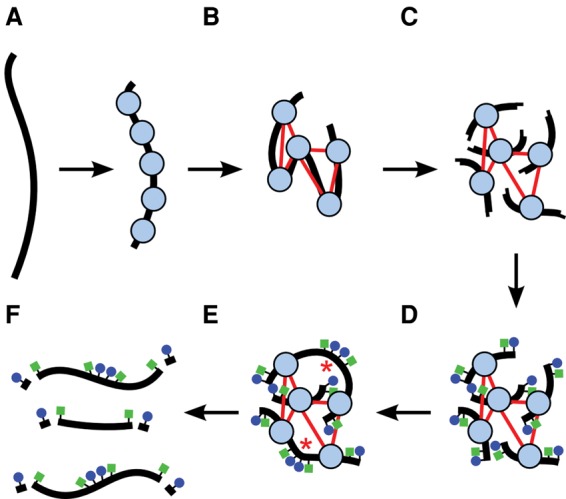
A diagram of a Chicago library generation protocol. (*A*) Chromatin (nucleosomes in blue) is reconstituted in vitro upon naked DNA (black strand). (*B*) Chromatin is fixed with formaldehyde (thin red lines are crosslinks). (*C*) Fixed chromatin is cut with a restriction enzyme, generating free sticky ends (performed on streptavidin-coated beads; data not shown). (*D*) Sticky ends are filled in with biotinylated (blue circles) and thiolated (green squares) nucleotides. (*E*) Free blunt ends are ligated (ligations indicated by red asterisks). (*F*) Crosslinks are reversed and proteins removed to yield library fragments, which are then digested with an exonuclease to remove the terminal biotinylated nucleotides. The thiolated nucleotides protect the interior of the library fragments from digestion.

We extracted 5.5 μg of high-molecular-weight DNA for Chicago libraries (in fragments of ∼150 kbp using the Qiagen HMW DNA kit and in fragments of ∼500 kb with agarose gel plug extraction) from the human cell line GM12878 and from the blood of a wild-caught American alligator (Supplemental Fig. S1). We reconstituted chromatin by combining the DNA with purified histones and chromatin assembly factors. Ordered chromatin assembly was confirmed by partial MNase digestion and gel electrophoresis (Supplemental Fig. S2). The reconstituted chromatin was then fixed with formaldehyde, and Chicago libraries were generated (Fig. 1 and Methods).

For the human GM12878 sample, we generated three Chicago libraries. Two libraries were generated from DNA with an average size of 150 kb and using either the restriction enzyme MboI (library “L1”) or MluCI (“L2”). The ultra-high-molecular-weight (500 kb) library (“L3”) was created with MboI. These libraries were sheared to an average of 300–500 bp in size and ligated to adapters for sequencing on the Illumina HiSeq 2500 as paired 100-bp reads, generating 46 million pairs for L1, 52 million for L2, and 165 million read pairs for L3. For the American alligator (*Alligator mississipiensis*), we similarly constructed a single MboI Chicago library and sequenced it on a single lane, yielding 210 million read pairs.

**Figure 2.**
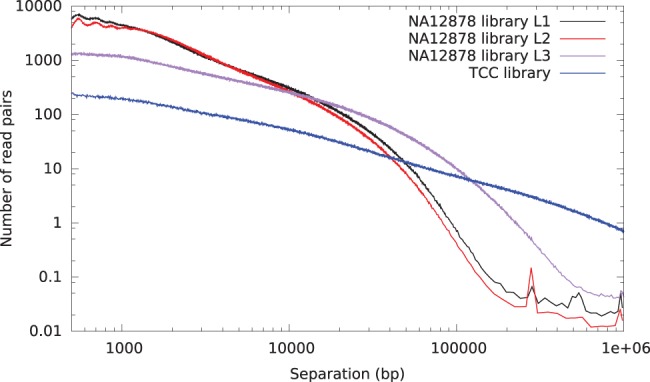
Histogram of read pair separations for several sequencing libraries mapped to hg19. (Black) Chicago library L1, prepared with MboI and 150-kbp input DNA; (red) Chicago library L2, prepared with MluCI and 150-kbp input DNA; and (violet) Chicago library L3, prepared with 500-kbp input DNA. A human Hi-C library (Kalhor et al. 2012) is shown in dark blue for comparison.

**Figure 3.**
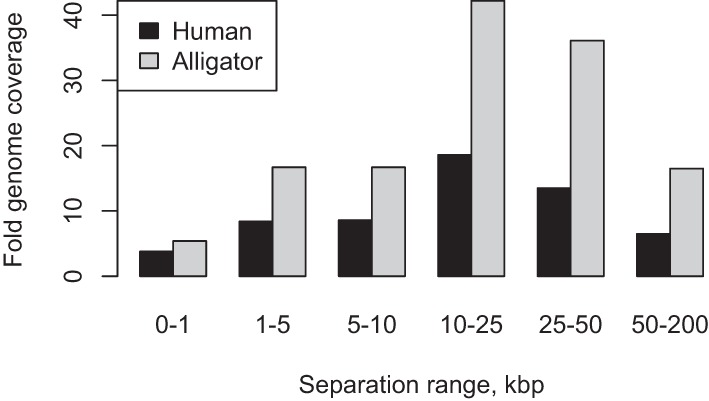
Genome coverage (sum of read pair separations divided by estimated genome size) in various read pair separation bins.

To determine the utility of these data for genome assembly and haplotype phasing, we aligned the GM12878 Chicago data to the reference human assembly, hg19 (Fig. 2). The Chicago libraries provided useful linking information for separations up to 150 kbp for L1 and L2 and up to 500 kbp for L3, consistent with the expected maximum size of input DNA fragments. By mapping these read pairs back to the reference human genome, we assessed the rate of background noise, defined for libraries L1 and L2 as reads pairs that map reliably to the genome but span distances >500 kbp or map to different chromosomes. For these libraries, we estimated the noise rate to be approximately one spurious link between unrelated 500-kbp genomic windows (mean of 0.97 such links). The linkage data span various size ranges. For illustration purposes, these data can be conceptually partitioned into various size bins based on the observed genomic distance between reliably mapped read pairs. Considered in this way, the single lane of sequencing from the GM12878 libraries provides linking information equivalent to 3.8×, 8.4×, 8.6×, 18.6×, 13.5×, and 6.5× physical coverage in 0- to 1-kbp, 1- to 5-kbp, 5- to 10-kbp, 10- to 25-kbp, 25- to 50-kbp, and 50- to 200-kbp bins, respectively, while for alligator the comparable coverage estimates were 5.4×, 16.7×, 16.7×, 42.2×, 36.1×, and 16.5× respectively (Fig. 3).

### Chicago data for genome scaffolding

We next determined the capability for Chicago data to aid the scaffolding of a previously described meraculous assembly of GM12878 that used 101-bp paired-end Illumina reads to yield 84× genomic coverage and a N50 of 33 kbp (Chapman et al. 2011; Simpson and Durbin 2012). First, we mapped the L1 and L2 Chicago read pairs to this initial assembly as described in the Methods. We found that 68.1% of read pairs mapped such that both forward and reverse reads had map-quality scores of 20 or greater and were thus considered uniquely mapping within the assembly and were not duplicates. Of these read pairs, 35.4% had forward and reverse reads that mapped to different contigs and were thus potentially informative for further scaffolding of the assembly. We also used the same Chicago data to scaffold a DISCOVAR assembly of 50× coverage in 250-bp paired-end reads (ftp://ftp.broadinstitute.org/pub/crd/Discovar/assemblies) with an initial scaffold N50 of 178 kbp (Weisenfeld et al. 2014). We found that 67.3% of L1 and L2 read pairs mapped to the DISCOVAR assembly with both forward and reverse reads having map quality scores of 20 or greater and were not duplicates. Of these reads, 26.5% mapped to different contigs.

We developed a likelihood model describing how Chicago libraries sample genomic DNA and integrated it with a software pipeline called “HiRise” for iteratively identifying and breaking misassemblies and for rescaffolding contigs based on Chicago links (Methods). We compared the completeness, contiguity and correctness at local and global scales of the resulting assembly to assemblies of rich WGS data sets, including extensive coverage in fosmid end pairs created by two of the leading WGS de novo assemblers: meraculous (MERAC) (Chapman et al. 2011) and ALLPATHS-LG (APLG) (Table 1; Supplemental Table S1; Gnerre et al. 2011). To avoid the arbitrary choices involved in constructing alignment-based comparisons of assembly quality, we based our comparison on the locations in the assembly of 25.4 million 101-bp marker sequences. Because the de novo assemblers report only a single haplotype at each locus, to avoid ambiguity we selected marker sequences that are a randomly selected subset of all distinct 101-bp sequences that occur exactly once in each haplotype of a diploid reconstruction of the GM12878 genotype (Rozowsky et al. 2011). In this way, these markers are likely single-copy, unique segments of the human genome that are homozygous in the individual we sequenced (GM12878). We then assessed each assembly by gauging the completeness and accuracy of these markers in each assembly versus the well-assembled human reference genome (Church et al. 2011).

**Table 1.**
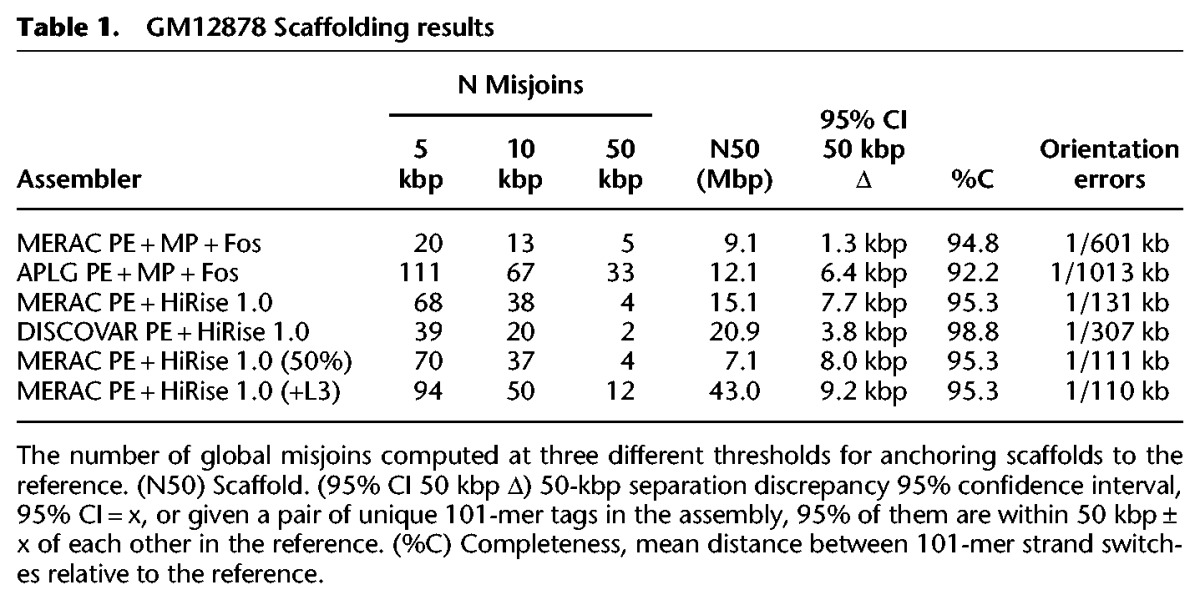
GM12878 Scaffolding results

### Long-range scaffolding accuracy

The genomic scaffolds that the HiRise pipeline produced were longer and had a lower rate of global misassemblies than the published meraculous and APLG assemblies, both of which rely on deep coverage in paired fosmid end reads. Table 1 shows the fraction of the total assembly found in scaffolds containing a misjoin. Misjoins were identified at three thresholds, as follows: A scaffold *s* is “anchored” to a chromosome *c* when all the marker 101-mers within some 5-, 10-, or 50-kb intervals on *s* are found on *c* in the reference. Scaffolds anchored to two or more chromosomes are classified as misjoined. To assess the completeness of each assembly, we computed the fraction of marker 101-mers present.

Because the DNA ligation events that create Chicago pairs are not constrained to produce read pairs of defined relative strandedness, contig relative orientations during scaffolding must be inferred from read density information. As a result, the Chicago HiRise scaffolds have a higher rate of scaffolding orientation errors. For each of the four human genome assemblies compared in Table 1, we counted the number of pairs of consecutive 101-mers along the scaffold that map to the same reference chromosome but with incongruent orientation, indicating a strand switch in the assembly, and report the mean density of such errors on the genome. Similarly, the broad range of read pair separations in the Chicago library can lead to more uncertainty in the estimation of gap sizes. To assess the impact of this on the assemblies, we identified pairs of marker 101-mers that were separated by *s*_*a*_ between 49.5 and 50 kb in each assembly, and examined their separations *s*_*r*_ in the reference genome; we report in Table 1 the minimum separation discrepancy *x* such that |*s*_*a*_ – *s*_*r*_| < *x* for 95% of the sample. The sample sizes were 458,966 and 478,494 marker pairs for the MERAC/HiRise and DISCOVAR/HiRise assemblies, respectively. The Supplemental Material includes a graphical depiction of all MERAC/HiRise scaffold misjoins.

To assess the effect on scaffolding quality on the quantity of Chicago data generated, we used a 50% subsample of the L1 and L2 libraries to scaffold the 30-kb N50 meraculous assembly and found that this reduced the scaffold N50 to 7.1 Mb (a 53% reduction) with a comparable number of misjoins. When we increased the coverage in Chicago data to 1.7× the original physical coverage with the addition of the L3 library, the scaffold N50 increased nearly threefold to 43 Mb, while the number of misjoins counted at the most sensitive of the thresholds that we used increased by 38% to 94 (Table 1).

### Improving the alligator assembly with Chicago data

To further assess the utility of Chicago data for improving existing assemblies, we generated a single Chicago library for the American alligator and mapped these data to a de novo assembly (N50 81 kbp) created using publicly available data (Green et al. 2014), and applied the HiRise scaffolding pipeline. The resulting assembly had a scaffold N50 of 10.3 Mbp. To assess the accuracy of these scaffolds, we aligned a collection of 1485 previously generated (Shedlock et al. 2007) bacterial artificial chromosome (BAC) end sequences to the assembly. Of those, 1298 pairs were uniquely aligned by GMAP (Wu and Watanabe 2005) with 90% coverage and 95% identity to the genome assembly and the HiRise scaffolded version. In the input assembly, 12.5% of the BAC end pairs were captured in the same scaffold with the expected orientation and separation. In the HiRise assembly, 96.5% of the BAC end pairs were aligned in the same scaffold with 98.1% of the BAC end pairs on the same scaffold in correct relative orientation. Five (0.39%) BAC end pairs were placed on the same scaffold but at a distance significantly larger than the insert size, and 14 (1.08%) were placed on separate scaffolds but far enough from the edge of the scaffold that the distance would be larger than the insert size, suggesting a global density of misjoins of fewer than one per 8.36 Mbp of assembly.

### Identification of structural variants

**Figure 4.**
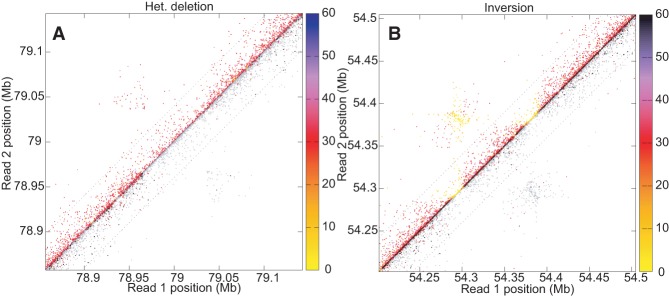
The mapped locations on the GRCh38 reference sequence of Chicago read pairs are plotted in the vicinity of structural differences between GM12878 and the reference (*A*, deletion; *B*, inversion). Each Chicago pair is represented both *above* and *below* the diagonal. *Above* the diagonal, color indicates map quality score on the scale shown; *below* the diagonal, colors indicate the inferred haplotype phase of Chicago pairs based on overlap with phased SNPs, with read pairs of unknown haplotype origin shown in gray.

Mapping paired sequence reads from one individual against a reference is the most commonly used sequence-based method for identifying differences in genome structure like inversions, deletions, and duplications (Tuzun et al. 2005). Figure 4 shows how Chicago read pairs from GM12878 mapped to the human reference genome GRCh38 reveal two previously identified structural differences, and illustrates how the variant haplotype phase can be inferred. Supplemental Figures S3 and S4 show schematically the expected read mapping distributions. Because GM12878 derives from an individual that has been trio-sequenced, gold-standard haplotype phase information is available to check the accuracy of Chicago phasing information. Read pairs that are haplotype informative and that span between 10 and 150 kbp are 99.83% in agreement with the known haplotype phase for GM12878. This allows confident assignment of variant allele phase based on read mapping.

To estimate the sensitivity and specificity of Chicago data for identifying structural differences, we tested a simple maximum likelihood discriminator (Methods) on simulated data sets constructed to simulate the effect of heterozygous inversions. We constructed the test data by randomly selecting intervals of a defined length *L* from the mapping of our Chicago GM12878 reads to the GRCh38 reference sequence, assigning each Chicago read pair independently at random to the inverted or reference haplotype, and editing the mapped coordinates accordingly. Nonallelic homologous recombination is responsible for much of the structural variation observed in human genomes, resulting in many variant breakpoints that occur in long blocks of repeated sequence (Kidd et al. 2008). We simulated the effect of varying lengths of repetitive sequence surrounding the inversion breakpoints by removing all reads mapped to within a distance *W* of them. In the absence of repetitive sequences at the inversion breakpoints, we found that for 1-, 2-, and 5-kbp inversions, respectively, the sensitivities (specificities) were 0.76 (0.88), 0.89 (0.89), and 0.97 (0.94), respectively. Simulating 1-kbp regions of repetitive (unmappable) sequence at the inversion breakpoints, the sensitivity (specificity) for 5-kbp inversions was 0.81 (0.76).

## Discussion

We have described an in vitro method for generating long-range mate-pair data that improves the scaffolding of de novo assembled genomes from high-throughput sequencing data. This approach has several advantages over existing methods.

First, Chicago library construction requires no living biological material, namely, no primary or transformed tissue culture or living organism. The libraries described here were each generated from 500 ng to 5 μg of input DNA. Furthermore, although the in vitro chromatin reconstitution is based on human histones and chromatin assembly factors, DNA samples from a wide variety of plants, animals, and microbes can be substrates for in vitro chromatin assembly using the protocol described. Our production facility has successfully generated Chicago libraries from several plants, prokaryotes, and vertebrate and invertebrate animals. As expected for histones that indiscriminately bind DNA, the chief considerations for successful in vitro chromatin assembly are the purity of the DNA and not its biological source.

Second, because Chicago data are generated from proximity ligation of chromatin assembled in vitro rather than chromatin obtained from in vivo sources, there is no confounding biological signal (e.g., telomeric clustering or chromatin looping) to potentially confuse the assembly. As expected for in vitro assembled chromatin, we find a low background rate of noise and a virtual absence of persistent and spurious read pairs. Supplemental Figure S3 shows a comparison of the distribution of equal numbers of Chicago and Hi-C pairs in a 4-Mb region of the human genome.

Third, in contrast to in vivo Hi-C methods, the maximum separation of the read pairs generated is limited only by the molecular weight of the input DNA. This has allowed us to generate contiguous scaffolding of vertebrate genomes using just short fragment Illumina sequence plus Chicago libraries. To date, high-quality scaffolding based on in vivo Hi-C libraries has started from assemblies with an order of magnitude more scaffold contiguity than the 30-kbp N50 input contigs successfully scaffolded by Chicago HiRise. Nevertheless, it remains the case that the Chicago libraries we have generated do not span all difficult-to-assemble regions. Centromeres, for example, are typically >1 Mb in size in the human genome. The smallest centromere in the human genome is on the Y Chromosome and is estimated to span 300 kb (Miga et al. 2014). In our experience, we can reliably prepare DNA spanning up to ∼150 kb from commercially available high-molecular-weight kits. DNA extraction and preparation methods that recover clean DNA of larger sizes have been described. However, we find that the high-molecular-weight kits provide DNA that allows for an attractive combination of speed, reliability, flexibility in input sample requirements, and performance in the Chicago protocol.

Fourth, these libraries eliminate the need for creating and sequencing a combination of long-range “mate-pair” and fosmid libraries and do not require the use of expensive, specialized equipment for shearing or size-selecting high-molecular-weight DNA that is normally required to create such libraries. Our approach thus greatly simplifies genome assembly as a single library is generated that spans short, medium, and long-range connectivity—up to the size of the input DNA.

In summary, we have presented simple DNA library construction and associated bioinformatic methods that generate significantly longer-range genome assembly scaffolds than existing methods. Furthermore, we have demonstrated the usefulness of our data for the discovery of structural genome variation. Our methods and results mark a substantial step toward the goal of accurate reconstruction of full-length haplotype-resolved chromosome sequences with low effort and cost.

## Methods

### DNA preparation

DNA was extracted with Qiagen blood and cell midi kits according to the manufacturer's instructions. Briefly, cells were lysed and centrifuged to isolate the nuclei. The nuclei were further digested with a combination of Proteinase K and RNase A. The DNA was bound to a Qiagen genomic column, washed, eluted and precipitated in isopropanol, and pelleted by centrifugation. After drying, the pellet was resuspended in 200 μL TE (Qiagen).

### Chromatin assembly

Chromatin was assembled overnight at 27°C from genomic DNA using the Active Motif in vitro chromatin assembly kit. Following incubation, 10% of the sample was used for MNase digestion to confirm successful chromatin assembly.

### Biotinylation and restriction digestion

Chromatin was biotinylated with iodoacetyl-PEG-2-biotin (IPB). Following biotinylation, the chromatin was fixed in 1% formaldehyde for 15 min at room temperature (RT) , followed by a quench with twofold molar excess of 2.5 M glycine. Excess IPB and cross-linked glycine were removed by dialyzing chromatin in a Slide-A-Lyzer 20-KDa MWCO dialysis cassette (Pierce) against 1 liter of dialysis buffer (10 mm Tris-Cl at pH 8.0, 1 mM EDTA) for a minimum of 3 h at 4°C . Subsequently, the chromatin was digested with either MboI or MluCI in 1× CutSmart for 4 h at 37°C. The chromatin was again dialyzed in a 50-KDa MWCO dialysis Flex tube (IBI Scientific no. IB48262) for 2 h at 4°C and then again with fresh buffer overnight to remove enzyme as well as short, free DNA fragments.

Dynabead MyOne C1 streptavidin beads were prepared by washing and resuspending in PBS + 0.1% Tween-20, before adding to chromatin and incubating for 1 h at RT. The beads were then concentrated on a magnetic concentrator rack, before being washed, reconcentrated, and resuspended in 100 μL 1× NEBuffer 2.

### dNTP fill-in

To prevent the labeled dNTPs (Fig. 1) from being captured during the fill-in reaction, unbound streptavidin sites were occupied by incubating beads in the presence of free biotin for 15 min at RT. Subsequently, the beads were washed twice before being resuspended in 100 μL 1× NEBuffer 2.

Sticky ends were filled in by incubating with dNTPs, including a-S-dGTP and biotinylated dCTP along with 25 U of Klenow (no. M0210M, NEB) in 165 μL total volume at 25°C for 40 min. The fill-in reaction was stopped by adding 7 μL of 0.5 M EDTA. The beads were then washed twice in preligation wash buffer (PLWB; 50 mM Tris at pH 7.4, 0.4% Triton X-100, 0.1 mM EDTA), before being resuspended in 100 μL PLWB.

### Ligation

Ligation was performed in at least 1 mL of T4 ligation buffer for a minimum of 4 h at 16°C . A large ligation volume was used to minimize cross-ligation between different chromatin aggregates. The ligation reaction was stopped by adding 40 μL of 0.5 M EDTA. The beads were concentrated and resuspended in 100 μL extraction buffer (50 mM Tris-cl at pH 8.0, 1 mM EDTA, 0.2% SDS). After adding 400 μg Proteinase K (no. P8102S, NEB), the beads were incubated overnight at 55°C, followed by a 2-h digestion with an additional 200 μg Proteinase K at 55°C. DNA was recovered with SPRI beads at a 2:1 ratio, with a column purification kit, or with a phenol:chloroform extraction. DNA was eluted into low TE (10 mM Tris-Cl at pH 8.0, 0.5 mM EDTA).

### Exonuclease digestion

DNA was next digested for 40 min at 37°C with 100 U Exonuclease III (no. M0206S, NEB) to remove biotinylated free ends, followed by SPRI cleanup and elution into 101 μL low TE.

### Shearing and library prep

DNA was sheared using a Diagenode Bioruptor set to “low” for 60 cycles of 30 sec on/30 sec off. After shearing, the DNA was filled in with Klenow polymerase and T4 PNK (no. EK0032, Thermo Scientific) for 30 min at 20°C. Following the fill-in reaction, DNA was pulled down on C1 beads that had been prepared by washing twice with Tween wash buffer before being resuspended in 200 μL 2× NTB (2 M NaCl, 10 mM Tris at pH 8.0, 0.1 mM EDTA at pH 8.0, 0.2% Triton X-100). Once the sample was added, the beads were incubated for 20 min at RT with rocking. Subsequently, unbiotinylated DNA fragments were removed by washing the beads three times before resuspending in low TE. Sequencing libraries were generated using established protocols (Meyer and Kircher 2010).

### Read mapping

Sequence reads were aligned with a modified version of SNAP (http://snap.cs.berkeley.edu/). Our modifications included masking out the base pairs that follow a restriction-enzyme junction (GATCGATC for MboI, AATTAATT for MluCI). Additionally, we removed the map quality penalty for read pairs that mapped to different scaffolds. PCR duplicates were marked using Novosort (http://www.novocraft.com/products/novosort/). Nonduplicate read pairs were used in analysis if both reads mapped and had a map quality score of 20 or greater.

### Ultra-high-molecular-weight Chicago library

Human GM12878 cells (Coriell) were grown in RPMI 1640 medium supplemented with 2 mM L-glutamine and 15% FBS using recommend growth conditions to a density of 5 × 10^6^ cells/mL. Cells were centrifuged and washed once with PBS and resuspended in ice-cold PBS at 1 × 10^8^ cells/mL. Cells were quickly warmed up to 37°C and then embedded in agarose by mixing 0.5 mL of the PBS suspension with 0.5 mL of 1.5% SeqKem LE agarose (Lonza) that had been first melted at 95°C followed by cooling and maintaining at 50°C. The agarose-cell suspension was rapidly aspirated in a 1-mL syringe and allowed to solidify for 60 min at 4°C. The agarose plug was unmolded from the syringe and incubated twice with 50 mL of lysis solution (2% sodium lauryl sarcosine, 0.4 M EDTA at pH 8.0, 0.5 mg/mL Proteinase K [recombinant PCR grade, Roche]) for 24 h at 55°C. The Proteinase K was then inactivated by incubating twice for 2 h with 50 mL of 0.1 mM PMSF at 4°C followed by at least 2 h in TE50 (10 mM Tris-Cl at pH 8.0, 50 mM EDTA at pH 8.0). The agarose plug was then incubated twice for 1 h with 50 mL 0.5× KBB buffer (Sage Sciences). The small DNA fragments and contaminants were removed by performing a 16-h electrophoresis using the 5- to 80-kb waveform type using the Pippin pulse electrophoresis system (Sage Science) by loading the agarose plug in a large preparative well. The DNA-embedded agarose plug was then cut in 1-mm slices (about six to 10 slices), and each slice was incubated twice for 1 h at 4°C with 400 µL Mg-free MboI buffer (10 mM Tris-Cl at pH 8.0, 100 mM NaCl) and for 1 h with 400 µL Mg-free MboI buffer containing 1 U MboI (Neb). Following the incubation with the MboI restriction enzyme, 5 µL of 1 M MgCl_2_ was added to each tube and incubated for 15 min at 4°C, then transferred for 30 min to 37°C, and then immediately transferred on ice and supplemented with 150 µL of 0.5 M EDTA (pH 8.0). The restriction enzyme was digested by adding 75 µL of 10% sodium lauryl sarcosine and 15 µL Proteinase K 20 mg/mL for 1 h at 37°C. The Proteinase K was inactivated by replacing the solution with 500 µL of 0.1 mM PMSF twice for 1 h at 4°C. The agarose slices were then transferred to a 15-kDa dialysis tube with a minimum amount of 0.5× KBB and subjected to 16 h of electrophoresis using the 5- to 430-kb waveform type on the Pippin pulse electrophoresis system followed by 10 min of electrophoresis with the opposite current direction. The dialysis tube was dialyzed three times for 1 h at 4°C against 1 liter of TE. The electroeluted DNA solution was recovered from the dialysis tube and stored at 4°C prior to chromatin assembly.

We generated a Chicago library from this very high-molecular-weight DNA and sequenced it on an Illumina HiSeq 2500 platform. Read processing and mapping were performed as described above.

### De novo assemblies

The human and alligator de novo shotgun assemblies were generated with meraculous 2.0.3 (Chapman et al. 2011) using publicly available short-insert and mate-pair reads (Simpson and Durbin 2012; Green et al. 2014). The alligator mate-pair reads were adapter-trimmed with Trimmomatic (Bolger et al. 2014). Some overlapping alligator short-insert reads had been “merged.” These were unmerged back into forward and reverse reads. The NA12878 APLG PE + MP + Fos assembly was downloaded from NCBI (BioProject accession PRJNA59877).

### Chicago HighRise (HiRiSE) scaffolder

#### Input preprocessing

To exclude Chicago reads that map to highly repetitive genomic regions likely to provide misleading links, we used the depth of aligned shotgun reads to identify problematic intervals. We used a double threshold strategy: Identify all intervals of the starting assembly with mapped shotgun read depth exceeding *t*_*1*_ that contain at least one base with a mapped read depth exceeding *t*_*2*_. In practice, we set *t*_*1*_ and *t*_*2*_ such that ∼0.5% of the assembly was masked. We also excluded all Chicago links falling within a 1-kbp window on the genome that is linked to more than four other input contigs by at least two Chicago links.

#### Estimation of likelihood model parameters

Several steps of the HiRise pipeline use a likelihood model of the Chicago data to guide assembly decisions or to optimize contig order and orientation within scaffolds. The likelihood function

$$L(l1,l2,g,o)=\frac{N!}{(N-n)!}(1-P_{0}{)}^{N-n}\prod_{i=1}^{n}\;f\;(d_{i})$$

gives the probability of observing the number *n* and implied separations of spanning Chicago pairs *d*_*i*_ between contigs 1 and 2, assuming the contigs have relative orientations o∈++,+−,−+,−− and are separated by a gap of length *g*. The function *f*(*x*) is the normalized probability distribution over genomic separation distances of Chicago read pairs and is assumed to have a contribution from “noise” pairs that sample the genome independently.

$$f\;(x)=\frac{\;p_{n}}{G}+(1-\;p_{n})f^{\prime }(x)$$

is represented as a sum of exponential distributions.

To obtain robust estimates of *N*, *p*_*n*_, *G*, and *f*′ (*x*) when the available starting assembly has limited contiguity, we first fixed an estimate of the product *N p*_*n*_, the total number of “noise” pairs by tabulating the densities of links (defined as *n*/*l*_1_*l*_2_) for a sample of contig pairs, excluding the highest and lowest 1% of densities, and setting $N_{n}=G^{2}\sum n_{ij}/\sum l_{i}l_{j}$, using the sum of the lengths of input contigs as the value of *G*. We then fit the remaining parameters in *Nf*(*x*) by least squares to a histogram of observed separations of Chicago read pairs mapped to starting assembly contigs after applying a multiplicative correction factor of $G{\left(\overset{N_{c}}{\underset{i=1}{\sum }}\text{min(}0,l_{i}-x)\right)}^{-1}$ to the smoothed counts at separation *x*.

#### Contig–contig linking graph construction

During the assembly process, the Chicago linking data were represented as a graph in which (broken) contigs of the starting assembly are nodes and edges are labeled with a list of ordered pairs of integers, each representing the positions in the two contigs of the reads from a mapped Chicago pair. The initial steps of scaffolding were carried out in parallel on subsets of the data created by partitioning the graph into connected components by excluding edges with fewer than a threshold *t*_*L*_ number of Chicago links. We chose *t*_*L*_ to be the lowest integer threshold that did not lead to any clusters comprising >5% of the input contigs.

#### Seed scaffold construction

The iterative phase of scaffold construction was seeded by filtering the edges of the contig–contig graph and decomposing it into high-confidence linear subgraphs. First, the contig–contig edges were filtered, and the minimum spanning forest of the filtered graph was found (see Edge Filtering below). The graph was linearized by three successive rounds of removing nodes of degree 1 followed by removal of nodes with a degree greater than 2. Each of the connected components of the resulting graph had a linear topology and defined an ordering of a subset of the input contigs. The final step in the creation of the initial scaffolds was to find the maximum likelihood choice of the contig orientations for each linear component.

#### Edge filtering

The following filters were applied to the edges of the contig–contig graph before linearization. Edges from “promiscuous” contigs were excluded. “Promiscuous” contigs were those for which the ratio of the degree in the graph of the corresponding node to the contig length in base pairs exceeds *t*_*p*_, or have links with at least *t*_*L*_ links to more than *d*_*m*_ other contigs. The thresholds *t*_*p*_ and *d*_*m*_ were selected to exclude ∼5% of the upper tail of the distribution of the corresponding value.

#### Contig orienting

Each input scaffold can have one of two orientations in the final assembly, corresponding to the base sequences of the forward and reverse, or “Watson” and “Crick,” DNA strands. The optimal orientations for the scaffolds in each linear string were found by dynamic programming using the following recursion relationship: In an ordered list of scaffolds of length *n*, the score of the highest-scoring sequence of orientation choices for the scaffolds up to scaffold *i*, such that scaffolds *i*−*k* −*k* to *i* have particular orientations *o*_*i*−*k*_,*o*_*i*−*k*+1_,…*o*_*i*_, is given by

$$\begin{array}{rl} S_{m} & \left(i,o_{i-k},o_{i-k+1},\ldots ,o_{i}\right)\\ & =\underset{o_{i-1-k}\in [+,-]}{\max}\left(S_{m}(i-1,o_{i-1-k},o_{i-k},\ldots ,o_{i-1})+\overset{\;j=i-1}{\underset{\;j=i-i-k}{\sum }}\log p(o_{j},o_{i})\right). \end{array}$$

Including links from contigs *k* steps back provided a significant improvement in orientation accuracy because small intercalated scaffolds might only have linking and therefore orientation information on one side, with important orientation information for the flanking scaffolds coming from links that jump over it.

#### Merge scaffolds within components

Contig ends were classified as “free” if they lie at the end of a scaffold or as “buried” if they were internal to a scaffold. For all pairs of contig ends within each connected component, the log likelihood ratio (LLR) score for joining them was computed with a “standard” gap size of *g*_*o*_. These candidate joins were sorted in decreasing order of score and evaluated according to the following criteria. If both ends are free and from different scaffolds, we tested linking the two scaffolds end-to-end. If one end is buried and the other is free and if the ends are from different scaffolds, we tested inserting the scaffold of the free end into the gap adjacent into the buried end. If one or both ends is buried and if the ends are on the same scaffold, we tested inverting the portion of the scaffold between the two ends. If both ends are buried and from different scaffolds, we tested all four ways of joining the scaffolds end-to-end. In all cases, the possible joins, insertions, and inversions were tested by computing the total change in LLR score by summing the LLR scores between all pairs of contigs affected by the change. If the change increased the LLR score, the best move was accepted.

#### Local order and orientation refinement

To refine both the local ordering and orientations of contigs in each scaffold, a dynamic programming algorithm was applied that slides a window of size *w* across the ordered and oriented contigs of each scaffold. At each position *i*, all the *w*!2^*w*^ ways of ordering and orienting the contigs within the window were considered, and a score representing the optimal ordering and orientation of all the contigs up to the end of the current window position that ends with the current O&O of the contigs in the window was stored. The scores of all “compatible” O&Os in windows at positions *i* − 1,*i* − 2,…*i* − *w*, and the scores of the extension of their orderings with the current O&O were used. Since *w*!2^*w*^ is such a steep function, the method is limited in practice to small values of *w*.

#### Iterative joining

After the initial scaffolds had been constructed within each connected component, the resulting scaffolds were returned to a single pool, and multiple rounds of end-to-end and intercalating scaffold joins were carried out. In each round, all pairs of scaffolds were compared, and likelihood scores were computed in parallel for end-to-end and intercalating joins. The candidate joins were then sorted, and nonconflicting joins were accepted in decreasing order of likelihood score increase.

#### Break low-support joins within scaffolds

To identify and break candidate misjoins in the assembly, we used the likelihood model to compute the log likelihood change gained by joining the left and right sides of each position *i* of each contig in the starting assembly (i.e., the LLR,

$$L_{i}=\ln\frac{L(g=0)}{L(g=\infty )},$$

for the two contigs that would be created by breaking at position *i*). A robust version of the support score is made by virtually masking up to *n* bins of size *w* to the left or right of candidate breakpoints, such that that the bins contributing the most to the score are excluded. This score is less susceptible to misjoins mediated by repetitive sequences. When the resulting support scores fell below threshold values over a maximal internal segment of an input contig, we defined the segment as a “low support” segment. After merging low support segments lying within 300 bp of one another and excluding those within 1 kbp of a contig end, we either (1) introduced a break in the contig at the midpoint of the segment or (2) introduced, if the segment is longer than 1000 bp, breaks at each end of the segment.

#### Gap closing

HiRise can use paired-end shotgun reads to close some of the gaps of unknown sequence created when scaffolds are joined based on Chicago read pairs. Groups of reads localized by SNAP alignment to the vicinity of each such gap are passed to “marauder,” the gap-closing module of meraculous, which returns a gap-closing sequence when a unique closure can be inferred by local *k*-mer walking.

## Data access

The HiRise scaffolder source code used here (version 0.75) is available in the Supplemental Material and hosted on GitHub at https://github.com/DovetailGenomics/HiRise_July2015_GR. The Chicago reads for human L1, L2, and L3 have been submitted to the NCBI Sequence Read Archive (SRA; http://www.ncbi.nlm.nih.gov/sra/) under accession numbers SRR2911057, SRR2911058, and SRR2911066, respectively; the Chicago reads for alligator, under accession number SRR2911055. Genome assemblies have been submitted to BioProject under the following accession numbers: PRJNA306147 (NA12878 MERAC PE + MP + Fos), PRJNA305645 (NA12878 MERAC PE), PRJNA301471 (MERAC PE + HiRise 1.0), PRJNA305644 (NA12878 DISCOVAR PE + HiRise 1.0), PRJNA305314 (MERAC PE + HiRise 1.0%–50%), PRJNA305315 (MERAC PE + HiRise 1.0 + L3), PRJNA305633 (DISCOVAR PE), PRJNA305630 (Alligator MERAC PE + HiRise 1.0), and PRJNA301461 (Alligator MERAC PE).

### Competing interest statement

The authors have applied for patents on technology described in this manuscript, and Dovetail Genomics LLC is established to commercialize this technology. R.E.G. is Founder and Chief Scientific Officer of Dovetail Genomics. D.H. and D.S.R. are members of the Scientific Advisory Board.
